# ‘Peace-kept’ urbanism: Ephemerality and endurance in eastern DRC

**DOI:** 10.1177/00420980241308111

**Published:** 2025-01-24

**Authors:** Maren Larsen

**Affiliations:** University of Basel, Switzerland

**Keywords:** camps, Congo, peacekeeping, temporality, urbanism, 营地, 刚果, 维和, 时间性, 城市化

## Abstract

This paper opens up and departs from United Nations peacekeeping camps in the city of Goma, Democratic Republic of the Congo, to grapple with questions around urbanism’s temporariness and permanence. Inspired by literature from southern urbanism and camp urbanism that focuses on temporal aspects of the built environment, I trace the various spatio-temporal horizons through which peacekeeping camps come in and out of being. Honing in on a particular moment of the United Nations Organization Stabilization Mission in the Democratic Republic of the Congo that registers both its extendedness and acknowledgement of an eventual end, four empirical examples illustrate the overlapping temporal logics shaping the spaces of these contingent camps. I trace these logics in ways that can be analytically useful to understanding how urbanism emerges in the continuous re-making of human settlements between now and later, as well as between the city and elsewhere. In doing so, I develop the notion of ‘peace-kept’ urbanism to account for dwelling arrangements in places where there is peacekeeping, marked by both ephemerality and endurance and fluctuating in conjunction with multiple spatial and temporal horizons.

## 摘要

本文以刚果民主共和国戈马市的联合国维和营地为背景，探讨城市化的临时性和永久性问题。南方城市化和营地城市化关注建成环境的时间特性，受相关文献的启发，本文追踪了维和营地出现和消失的各种时空界域。我们通过四个实例，对联合国组织刚果民主共和国稳定特派团的某个特定时刻进行了研究。这一时刻既体现了其延伸性，也标志着其最终将结束。这四个实例阐释了塑造这些临时营地空间的重叠的时间逻辑。我们以有利于分析的方式追踪这些逻辑，有助于理解城市化如何在现在和将来，在城市和其他地方，在不断重塑人类住区的过程中出现。为此，我们提出了“维和”城市化的概念，以解释维和地区的居住安排，这种安排既短暂又持久，并与多个空间和时间界域一起波动。

## Introduction

In the late 2010s, the city of Goma, together with the adjacent towns of Mubambiro and Munigi, hosted the highest concentration of bases accommodating military contingents serving in the United Nations peacekeeping mission in the Democratic Republic of the Congo (DRC). Between 2017 and 2019, contingents of varying troop strengths from eight different countries in the Global South were accommodated across 22 different sites generally referred to by their military peacekeeper inhabitants as company operating bases (COBs) or more simply, camps. The geography of peacekeeping camps in and around Goma is ever-changing, shifting in relation to strategic and security considerations, operational demands, economic efficiencies, environmental sustainability and political negotiations. Peacekeeping camps in and around Goma distinguish themselves from civilian compounds in the city by providing nightly accommodation for military contingents. While they serve as the primary living spaces for military men and women of a single national group, different security configurations monitor access to these camps by mission personnel of different nationalities, Congolese contractors and civilians^1^ and, between 2017 and 2019, an American anthropologist.^2^ Stepping inside and opening up these largely ignored and overlooked urban spaces, this article unpacks the temporary and permanent architectures made for, with and by the military contingents serving the United Nations Organization Stabilization Mission in the Democratic Republic of the Congo, more commonly referred to as MONUSCO. I argue that peacekeeping camps’ situation between an enduring presence and a planned expiration unsettles several taken-for-granted assumptions about where and when urbanism and urbanity can emerge.^3^

Based on 15 months of fieldwork and participatory observation regarding the everyday lives of UN peacekeepers, I seek to bring forth examples of how the city’s peacekeeping camps are built through, with and against time. Two particular temporalities provide important context for investigating peacekeeping camps: the first is the short-term nature of individual contingent deployments (lasting, on average, one year for the contingents in this study). The second is the protracted nature of violent conflict in eastern DRC and repeated renewals of MONUSCO’s mandate. The analytical space that this latter situation opens up is one within a ‘no peace, no war’ continuum – one on which external interventionism is both predicated and depends for its continuation (Richards, 2005). While field tenures and peace tenures are generally thought of as mutually exclusive deployments for peacekeepers from countries with active conflicts, Goma’s peacekeeping camps and the activities and possibilities within them combine elements of a combat field station and peace station, as the empirics brought forth here illustrate. From Goma, peacekeepers adopt an operational concept known as ‘protection through projection’ for territories further afield while the city itself benefits from ‘protection through presence’ of a high concentration of troops. Both peacekeepers themselves and subnational statistical analyses confirm that peacekeeper presence is an effective deterrent to armed group activity (Personal Communication 8 February 2019; Fjelde et al., 2019).

However, peacekeepers’ presence does not make Goma an entirely ‘peace-kept’ place, hence the proposal here to problematise and cast doubt upon the term by placing it in quotation marks. As Büscher (2020) argues, Goma acts as a safe haven relative to wider geographies of violence and insecurity but, as shall be problematised later, does so for an array of actors that includes armed groups and state actors that commit human rights violations. Moreover, as Hendriks and Büscher (2019) highlight, broader war dynamics ‘reinforce the violent nature of urban crime’– crime being something that peacekeepers’ understand as a ‘law and order problem’ beyond their mandate of intervention (Larsen, 2020, forthcoming). Adopting the perspective of the military peacekeepers present in this study, the ‘at war, at peace’ nexus in which their everyday lives unfold begets another productive tension that is at the heart of this article: the competing *horizons* that impel peacekeeping camps to endure and expire. Following Vigh (2008: 16): ‘The concept of horizon becomes analytically interesting in this perspective as it contains both a temporal and spatial dimension.’ I find productive resonance between the anthropological deployment of temporal and spatial horizons and Massey’s insistence on actively thinking about space *together with* time to illustrate the multiple determinants and outcomes of peacekeeping camps’ unique temporal conditions (Massey, 2005, emphasis added).

I focus on the year 2019 as a particular moment in mission time – one that simultaneously registered the protractedness of peacekeeping in the Democratic Republic of Congo and brought peacekeepers’ eventual and final departure into more immanent view. MONUSCO’s predecessor MONUC was established in 1999 as a means of monitoring the Lusaka Ceasefire Agreement, making 2019 the 20th anniversary of a multi-national peacekeeping presence in the DRC. In addition to marking the endurance of a mission and its shortcomings in stabilising the country’s security situation, 2019 was also the year in which the United Nations Security Council requested an external assessment of outstanding challenges to peace and the articulation of an exit strategy (Mahmoud, 2019). The development of a drawdown strategy was common knowledge among the officer ranks present in Goma at the time of my fieldwork and was increasingly raised in conversation to explain a number of spatial policies affecting various battalions. As the empirics presented in this article attest, however, the built environments of the camp as they evolved in 2019 at once adhered to logics of an eventual withdrawal of the mission from Congolese territory and defied them.

Below, I situate the study of peacekeeping camps within urban studies literature that deals with aspects of temporariness in the built environment, connecting some of these ideas to the burgeoning literature within this field that focuses on camp spaces specifically. Firstly, I want to challenge the notion that urban settlements are created or consolidated solely through a drive towards permanence by bringing together literature from southern urbanism and the burgeoning field of camp studies (dominated by scholarly inquiries into refugee camps).^4^ Rather than reinforcing a dualism between the temporary and the permanent that tethers urbanism to some semblance of permanence, scholarly literature in southern urbanism and camp studies problematises this dichotomy – as temporary camp spaces in particular have long been theorised with analogies and comparisons to cities and urban processes (Agier, 2002; Jansen, 2019; Rawlence, 2017). Secondly, I trace the logics that impel the peacekeeping camps’ endurances and ephemeralities, as well as peacekeepers’ experiences of camp time as flexible durations entangled with spaces beyond its confines (Hailey, 2008). Key to understanding the peacekeeping camp’s inherent un-settledness and variable temporariness and permanence is an acknowledgement of the ways in which it exceeds itself and is shaped by considerations of various elsewheres, including the city immediately beyond its gates, the areas of operations to which peacekeepers are deployed, and the places they come from and to which they plan to return. These connections, I argue, shape the sociality and built environment of peacekeeping camps themselves and of the city that hosts them, begetting a particular type of ‘peace-kept’ urbanism.^5^ Tracing the spatio-temporal tensions making the camp serves to introduce a mode of making that returns our attention to the perpetual becoming of urban space and to the ways that people in motion make and re-make settlements between then, now and later, and between here, there and elsewhere.

## Problematising permanence from the city to the camp

Planning practice and theory continue to overlook or devalue the place of the provisional in the production of urban spaces and lifeworlds despite attempts by scholars to recover its significance and possibilities. Scholars and theorists of southern urbanisms writing in English have been particularly prolific in highlighting temporary installations’ ability to offer alternatives to neoliberally-informed plans and policies and generate urban modes of being in their own right (Andres, 2013; Guma, 2021; Simone, 2018). For instance, from the Kibera settlement in Nairobi, Guma (2021) illustrates how the increased permanence brought about by a slum upgrading project precluded several livelihood strategies and their attendant socialities that residents had come to value and depend on – making the new tenement housing less desirable for some.

The Southern critique’s power vis-à-vis temporality pivots upon its insistence that interstitial, makeshift and ephemeral urbanisms are not lesser urbanisms. As Mehrotra (2019) reminds us through his analysis of Kumbh Mela’s mode of assembling and disassembling an ephemeral megacity, urbanism can be an ‘elastic condition’. That elasticity allows us to imagine a planning politics that foregrounds thinking about eventual disassembly, a sustainable re-absorption of materials, and present possibilities to adjust to volatile circumstances and environments through temporary uses. As a mode of making taken up overwhelmingly by people in and from the Global South, ‘peace-kept’ urbanism, I contend, joins this rich literature of southern urban practice.

Examples of urban forms and practices that defy a quest towards permanence are not only a hallmark of southern urbanism, but are visible as well in light of increasing austerity and economic crisis in EuroAmerican contexts (Madanipour, 2017; Stevens and Dovey, 2023; Tonkiss, 2013). As the scholars studying these temporary interventions attest, interim uses and transitory occupations can work to disrupt neoliberal politics of urban development but they can also end up following entrepreneurial logics when made a part of regeneration efforts or creative city branding (Bragaglia and Caruso, 2022; Groth and Corijn, 2005; Madanipour, 2018; Tonkiss, 2013). Importantly for my argument, urban temporariness, when understood as not only expressing a lack or deficiency but as offering agentive alternatives, help us critique assumptions about the primacy of more seemingly stable, consolidated or durable urban configurations and linear narratives of development that make permanence synonymous with improvement or the ability of urban settlements to ever be complete.

Southern urban scholarship is not the only scholarly literature to consider and valorise the temporary arrangements that make cities into social, cultural and architectural urban spaces. Studies of camp settlements, particularly since what Jansen (2016) has called the ‘modest “urban turn” in refugee studies’ have provided particularly thoughtful meditations on the multiple temporalities shaping camp urbanism beyond a drive towards permanence. Literature on the refugee camp continues to dominate urban scholarship of camp space and has been prolific in bringing to the fore the entangled logics and politics of temporality and permanence that have come to characterise their inhabitants’ experiences of space (Abourahme, 2011; Abourahme and Hilal, 2012; Agier, 2008; Feldman, 2015; Herz, 2013; Herz and ETH Studio Basel, 2013; Hilal and Petti, 2018; Ramadan, 2013). The temporality of the refugee camp has consistently been theorised as existing between the permanent and the temporary, begetting variable and inherently unstable subjectivities. Abourahme (2020) calls this in-betweenness a ‘political–historical threshold’, the crossing of which, in the case of Palestinian refugees, entails ‘opening up a temporality between the *permanence* of the built (camp) and the *temporariness* of the (a)political condition (refugeehood)’ (p. 41, emphasis in original). Staying with the Palestinian example, Alessandro Petti and Sandi Hilal propose *Permanent Temporariness* as a way of framing their collective experimentations and engagements with the architecture of the camp. Their work most pointedly revalorises the temporariness of Palestinian refugee urbanism as a political demand for the right of return. As Hilal reminds us, ‘the colonial project has worked to convince people that the only valid and valuable culture is the permanent one’, while Petti adds that ‘a common struggle could be to destabilise the binary notion that rights and a good life can only be obtained with permanency and that precarity and exploitation are brought about by temporariness’ (Hilal and Petti, 2018: 55). Manuel Herz’s study of Sahrawi refugee camps in Algeria comes to a similar conclusion, theorising the extended inhabitation of the tent as a proclamation of the right of return to Western Sahara, *as well as* a right to recover architectural cultures associated with Sahrawis nomadic livelihood strategies (Herz and ETH Studio Basel, 2013).

The political condition of Palestinian and Sahrawi refugees, exiled by the Israeli and Moroccan occupations respectively, produces a particular politics of inhabitation and temporal liminality in the refugee camp that cannot easily be extended to other experiences of camp settlements with different political–historical thresholds. The forced displacement of refugees and voluntary deployment of peacekeepers underscore markedly different political conditions of encampment. The contribution that refugee camp literature offers for scholars interrogating different kinds of temporary dwelling arrangements is to adopt an analytic that recognises residents’ imaginations of the future *and* the past to read the claims embedded in more ephemeral architectures.

Inspired by literature on the refugee camp that critically interrogates the temporal calibrations and defiances of the built environment, the following pages seek to unpack another kind of camp entirely, to highlight the types of considerations and the multiple temporalities and elsewheres implicated in contingent politics of inhabitation (pun intended). UN peacekeeping bases have largely been overlooked in urban studies’ considerations of camp space, yet they are an increasingly present feature of the post-millennial, built environment in places experiencing conflict in the Global South and in sub-Saharan Africa in particular.^6^ In 2016 in sub-Saharan Africa alone, there were 170 cities where UN peacekeeping operations were taking place (Shoshan, 2016: 7). This figure comes from the exhibition ‘BLUE: Architecture of UN Peacekeeping Missions’ curated by Malkit Shoshan at the 15th Venice Architecture Biennale, which provides a notable exception to the lacuna of research on peacekeeping camps. Shoshan’s (2016: 13) analysis contends that peacekeeping camps are ‘islands’ in a wider aid ‘archipelago’ that have fragmenting effects on the surrounding urban areas (Duffield, 2012a). As the empirics presented here demonstrate, the temporalities at work in peacekeeping camps draw in and cast out multiple extra-camp dynamics and geographies that challenge a descriptive vocabulary that suggests isolation.

**Figure 1.**
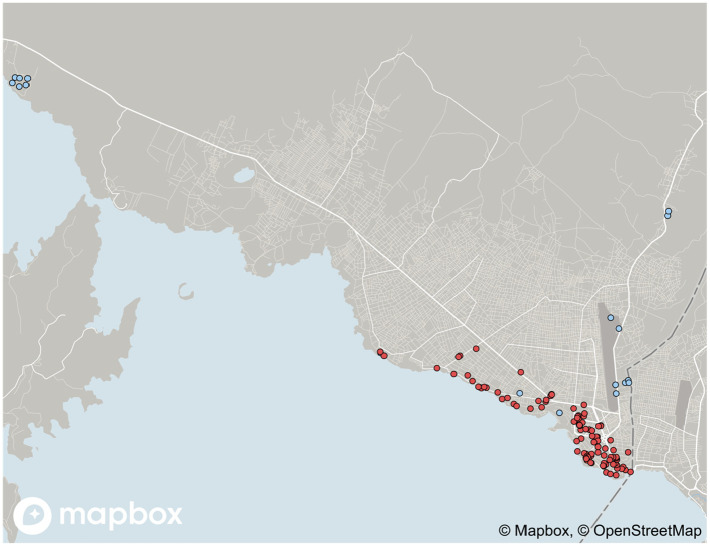
Spatial footprint of peacekeeping camps (light/blue) and international humanitarian offices and compounds (dark/red) in and around Goma between 2017 and 2019. (Mapbox; OpenStreetMap; Data by author; Data by Living in Goma Blog by Timo Mueller.)

This review of relevant literature on temporariness and the provisional in southern urbanism and camp studies has notably bypassed a more sustained dialogue with humanitarian urbanism, which seeks to capture socio-economic and place-making processes that occur in places with an international humanitarian presence (including camp spaces) (Büscher and Vlassenroot, 2010, 2013; Cooley and Ron, 2002; Jansen, 2016, 2019; Potvin, 2013). The ‘peace-kept’ urbanism notion that this article elaborates foregrounds the urbanity that emerges specifically through the temporal calibrations of peacekeepers and their camps, rather than humanitarian actors.^7^ This distinction is necessary for several reasons. Firstly, temporariness and permanence does not feature as a key theme of this literature and its debates. Secondly, urban geographies of peacekeeping and humanitarian intervention differ due to different security and protection considerations (see Figure 1). Lastly, interrogating the lifeworlds of military men and women from the Global South demands a departure from explanations gleaned from the spaces, architectures and lifeworlds of foreign interventionism that tend to focus on white, EuroAmerican, civilian interveners (Autesserre, 2014; Duffield, 2012b; Smirl, 2015).

As the subsequent section illustrates, peacekeeping bases are charged with dynamic forces that impel a dialectic of endurance and ephemerality and are tethered to the political–historical thresholds or nexuses of conflict, the spaces in which they are situated, the places from whence peacekeepers come, and the multi-national peacekeeping project itself. Camps accommodating military contingents serving MONUSCO are particularly illustrative of the multiple temporalities and places that shape urban ways of life.

## The time of the peacekeeping camp: Between city, fields and homes elsewhere

Four particular events illustrate the ways that disparate place–times work upon the peacekeeping camp, bringing to the fore the constant dialectic of temporariness and permanence through which these camps are made, remade and unmade. The place–times highlighted through these events include the urban spaces across the spatial thresholds of the camp gates, the rural hinterlands to which peacekeepers are frequently deployed across the country, the places across the world that they call home and the urban futures of the camp sites themselves. While I am not denying that endurance over time can result in increasing material permanence and urbanisation of the built environment, the examples I bring to bear here are meant to illustrate ‘more-than-linear’ relationships that resist situating the temporary and the permanent at opposing ends of a given duration. These examples equally work to de-couple notions of instability and precarity tied to temporary dwelling arrangements and increased stability for inhabitants through more permanent configurations and architectures.

The first event worth highlighting is the disassembling of the Senegalese Formed Police Unit camp known as Camp *Jambaar*^8^ and the police units’ re-location from a site with concrete buildings to a camp marked by more modular living arrangements with shorter functional life spans. The abandonment of one camp and building of another highlights the intersection of an extended mission temporality and the work of time on urban land markets, as the endurance of the peacekeeping mission has made it difficult to justify recurrent expenses and has led to the formulation of several cost-saving measures. One key measure, formulated in 2018, sought to vacate private leases and maintain only the free land leases offered by the state and/or various religious organisations to save on rising rents in a rapidly urbanising city. For instance, MONUSCO’s LEVEL III hospital, which occupied a former hotel in the city centre before 2019, cost a reported $35,000 USD per month to rent for a mission that was looking to cut $2 million USD in leases alone during the course of 2018 (Personal Communication, MONUSCO Field Engineer, 23 October 2018).^9^ The former Camp *Jambaar* was located on land owned by a private coffee estate, thus prompting the mission to re-locate the Senegalese contingent to land governed by a free lease provided by the state (whose consent to a multi-national peacekeeping force, it is worth mentioning, is one of the principles of United Nations peacekeeping). The new camp was located across the road on a stretch of land owned by the state airways authority – its vacancy a product of its proximity to the airport runway, making it unsafe for permanent habitation. While the private site had several permanent structures that served as coffee processing infrastructures-turned-accommodations, mess halls and recreation spaces, Senegalese peacekeepers were moved to the new site and equipped with pre-fab modular units and relocatable structures. The contingents’ own experience of the re-location however was particularly positive. One of the main benefits of the new site was an increased sense of privacy for the women of the camp who were no longer accommodated in such close quarters to the men in the crowded, permanent building on the site of the former camp (Focus Group Discussion 25 October 2017).

New living arrangements and spatial configurations that were not more permanent but more temporary engendered several new practices that animated the Senegalese peacekeepers’ encounters with each other and the surrounding community in the new camp. In this new camp configuration, women were afforded their own enclosed recreation space in which they stayed up late watching TV, playing ludo and dancing *sabar*, strengthening their privacy, sisterhood and morale. Inhabitants of the camp also found themselves in closer proximity to the bustling Birere market and its street food vendors and began making a habit of purchasing roasted corn in the late afternoon to hold their stomachs over until the evening meal – even naming this practice a ‘*mboq* party’.^10^ Far from losing any of its urban character by vacating a permanent built environment, the new site and its more provisional location and amenities afforded more extended and private spatial arrangements within the camp and more proximate access to local vendors and services beyond its gates. Moreover, rather than reinforcing existing theorisations of peacekeeping camps as islands existing separately from their surroundings (Shoshan, 2016), this event highlights the ways in which the wider urban land market interacts with the economic decision making of the mission and how these decisions can breed new, albeit limited, socialities and proximities to the Congolese ‘host’ society.

The second series of events highlight the ways that provisional camp architectures, in their maintenance and incremental improvements by camp dwellers, provide an animating rhythm of everyday life in peacekeeping camps and work towards enhancing the quality of time spent at the base relative to the more mobile conditions of standing combat deployments further afield. Prior to occupying their current location along the Goma Airport runway,^11^ the Uruguayan battalion (known as URUBATT) was in constant motion across the DRC for about seven years, taking up positions relative to shifting geographies of conflict in Kalemie, Kindu, Kisangani and Kinshasa during the 2006 elections. In 2007/2008 they moved to their current location, a time that coincided with an eastward shift of Laurent Nkunda’s area of influence in North Kivu that heightened the threat of an attack on Goma from the north (Stearns, 2012). The relative settlement of the battalion in one place has engendered new ways of being together both between Uruguayan peacekeepers and the Congolese civilians living near their base, and among Uruguayan peacekeepers in-camp. The endurance of the camp has consolidated it as a space of opportunity (Jennings, 2015; Oldenburg, 2015), particularly for a small group of local youth, whose frequent interactions with Uruguayan peacekeepers have led to their fluency in Spanish, which they hope to leverage in Goma’s wider intervention market. Meanwhile, in camp and despite the planned temporariness of the camp, commanders passing through over the years have made the strategic yet arguably cost-inefficient decision to incrementally invest in the URUBATT camp’s continued improvement and beautification (Personal Communication, 7 August 2017).

As the mission has endured beyond the life-cycle of various materials used to build peacekeeping camps, decay and dilapidation have taken over many of their physical architectures. Commanders like Col. Martín (pseudonym) however seize upon the cyclical time of disrepair and repair in ways that value the camp’s enduring temporariness for the benefits of maintenance as a social practice in the camp (Personal Communication, 8 February 2019). From a commander’s perspective, maintenance work speeds up the time of the camp for troops, offering a structured activity that busies off-duty soldiers in an effort to ward off boredom and other psychological stressors that arise quicker in conditions of confinement and can work against the effectiveness of the warrior body. From the perspective of contingent troops themselves, the always provisional outcomes of that practice – a clean, smoothly running and aesthetically enjoyable URUBATT camp – are seen as something that can lift morale when juxtaposed against the taxing and dangerous living conditions of repeated field deployments (Personal Communication, 12 August 2017). The relative consolidation of the URUBATT camp offers a place to recharge between more arduous and temporary operations. Peacekeeping camp upkeep therefore, constantly re-makes the aesthetics of the camp while keeping infrastructures and materials functional but never completely fixed, so as to sustain the social practice of maintenance and beautification as a matter of peacekeepers’ own well-being and enjoyment of the environments in which they endure their peacekeeping tenure.

The act of maintaining physical infrastructures is one of the most important command tactics Col. Martín has at hand to care for and control his troops. The impetus to build new provisional infrastructures and continuously improve them can also come from the values and creative agencies of the soldiers themselves. The third event remains grounded in the URUBATT camp but highlights the place-times associated with peacekeepers’ more distant sites of departure and return – their countries of origin – and how these are expressed in the camp through the emergence and rapid consolidation of a particular architectures.

Around mid-2017, a curious practice began in URUBATT that initially confused the commanding officers of the camp: soldiers had made a habit of gathering in a small patch of grass behind the Officers Mess, particularly around midday, with their phones in their hands. The patch of grass was near one of the camp’s Wi-Fi routers, which gave troops a strong internet signal. The timing of the practice, which often came with a hot and sharp sun in Goma, coincided with the early hours of the morning in Uruguay when troops who wanted to catch a loved one before work or school could call. Soon after understanding the practice, URUBATT leadership approved the construction of a small gazebo to provide shade and seating near the router. As it came into being and began to be used, the gazebo began to express new meanings. According to a group of non-commissioned officers, a soldier entering the space of the gazebo can be a clear signal to others that they are homesick and want to be left alone, whether they manage to reach their loved ones on their phone or not. In Uruguayan military jargon, extreme homesickness can bring about a feeling known as the axe (*el hacha*), which can compromise troop health and welfare as it can make soldiers more prone to developing physical illnesses. As such, the installation of power outlets, landscaping, stone pathways and nearby murals stand as collective efforts to lift the spirits of those who retreat to this gazebo, aptly named the *Rincón del Hacha* (The Homesick Nook). Like other prominent sites in the camp, the contingent commissioned a Congolese woodworker who works out of a recycled container in the camp to create the signage for this new space. The *Rincón del Hacha*, while arguably one of the more materially durable architectures of the camp, is predicated on a practice of connecting to kith and kin elsewhere – a practice that connects them in the present to their past and future home. While the social practices that go on in this place connect troops to other times and places to which they plan to return, the nook’s increasing material permanence simultaneously defies mission logics of an eventual and total repatriation and dismantling. For the Force Commander, while repatriation is inevitable, the clearing of the camps is not, as the fourth illustration highlights.

‘My plan for the COBs is to hand over to the country’, the Force Commander tells me. In July 2019, as the exit strategy was being discussed and set in motion in Goma, I met with the highest-ranking military leader of MONUSCO to talk about what would happen to the peacekeeping camps amidst and after the withdrawal of the mission. By ‘the country’, he is referring to its security forces – primarily the *Forces Armées de la République Démocratique du Congo* (FARDC) and the *Police Nationale Congolais* (PNC). ‘They are not deployed as we would like to see them deployed. They do not have barracks. They are in huts or tents’ (Personal Communication, 24 July 2019). He sees the benefits of leaving the architectures and infrastructures of the peacekeeping camps for the national military in terms of comfort, training and discipline. Even the furniture will stay, he says. The camps themselves thus have an important role to play in MONUSCO’s support of security sector reform in the eyes of the Force Commander. As Pech et al. (2018) have identified, the Congolese state militarisation of the urban landscape in Goma is one of the dynamics driving intraurban growth and development. If the Congolese state does decide to install its military elements in former peacekeeping camp sites, then this has implications not only for the security situation in Goma, but presumably also its urbanisation pattern and quality of the built environment, illustrating how the spatio-temporal condition of peacekeeping camps exceed themselves to shape wider urban geographies of which they form part. Reporting in the spring of 2024 confirms that the peacekeeping camps handed over to the Congolese state and occupied by the FARDC in South Kivu have been plundered, run-down and inadequately provisioned (Murhabazi, 2024).

The day after my meeting with the Force Commander, I was in an Indian battalion camp to chat with Congolese language assistants and community liaison assistants about their work with military contingents in the mission. After the small focus group, I shared some of my initial impressions to Lt. Col. Dev (pseudonym) – the contingent commander with operational responsibility for the city of Goma itself. As we were discussing some of the key differences in rural and urban community engagement, Dev raised a significant yet controversial operational reality: ‘My armed groups are the PNC and the FARDC’ (Personal Communication, 25 July 2019).

Pairing this insight with the Force Commander’s re-use intentions for the camp, a key tension comes to light about the prospects for a more peaceful urban future in Goma with these enduring architectures. On the one hand, part of Goma’s reputation as a safe haven has been predicated on the concentration of Congolese army bases and police forces (Büscher, 2020). That same concentration, however has led to a pronounced military footprint in the city, contributing to the dilapidation of the urban architectural landscape (due to a lack of state resources) and heightened insecurity near the bases of Congolese security forces (Pech et al., 2018). Evidence from five MONUSCO base closures in Walikale and Masisi territories in 2017 also raises several concerns in terms of the security vacuum left in the wake of MONUSCO’s withdrawal. Persisting and worsening dynamics in rural towns experiencing mission base closures included increases in reports of weapon possession by civilians, banditry and the continued threats posed by armed groups of armed clashes, sexual violence, recruitment (including among children) and the reignition of ethnic tensions (UN Joint Human Rights Office/MONUSCO, 2018). The same base closure report cited several incidences of abuse committed by Congolese security forces themselves. As Lt. Col. Dev’s comment affirms, one of the dilemmas that peacekeepers stationed in Goma have faced has been navigating their cooperation with security forces who have often been the very perpetrators of human rights abuses (Hendriks and Büscher, 2019; Hirschmann, 2019; OHCHR, 2013; Pole Institute, 2017). Abuses committed by the military and police forces in the city have ranged from excessive force against protestors to extrajudicial killings, raising questions about whether or not the preservation and re-use of peacekeeping camps by national security forces can in fact contribute to a more peaceful future in Goma and in eastern DRC more generally.

## The multiple horizons of ‘peace-kept’ urbanism

The spatio-temporal horizons that shape the architecture and urbanism of the peacekeeping camps productively problematise an opposition of temporariness and permanence in our imagination of urbanism and highlight the dynamic currents that flow between camp spaces and the surrounding city. Empirical examples from across multiple peacekeeping camps in Goma and imaginations of their futures illustrate how the consolidation, re-location, demolition, repair, improvement and planned preservation of their built environments can emerge simultaneously depending on what horizon is being considered and by whom.

The value of thinking about the in-between temporal condition of the peacekeeping camp through the notion of horizons lies in the terms ability to distinguish various currents impelling both the camp’s endurance and its expiration. In Abourahme, Hilal, and Petti’s theorisations of the camp’s temporal liminality, the built environment of the refugee camp ‘picks a side’ between being qualified as temporary or permanent. For Abourahme (2020 ), the built environment is the permanence juxtaposed against the temporariness of refugeehood, while for Hilal and Petti (2018), the built environment is viewed as temporary to serve refugee’s politics and preserve their right of return. Following the spatial and temporal horizons of the peacekeeping camp – to urban land markets in Goma, to past and future field deployments in the country, and across time zones and geographies back to peacekeepers’ countries of origin – offers the ability to account for the multiple intersections of social, political, economic and geographic considerations that impel architectural changes to the peacekeeping camp. The horizons of the peacekeeping camp do not pick a side or allow for a mere acknowledgement of a liminal or in-between condition of the camp, but rather offer a means to account for the disparate place–times connected to the permeance and temporariness of places that host peacekeepers. Doing so helps to counter understandings of these spaces as detached from their surroundings and highlights new attachments across wider geographies (Shoshan, 2016), further evidencing the inadequacy of understanding peacekeeping camps as part and parcel of Peaceland – a metaphorical bubble disconnected from the lifeworlds of the ‘host’ society (Autesserre, 2014).

The multiple spatio-temporal horizons of military peacekeeping that get refracted through the built environments of contingent camps and the cities that host them demands a concept-term that captures the range of socio-spatial formations – in their permanence and temporariness – that emerge in places inhabited by peacekeepers. My proposal to develop a notion of ‘peace-kept’ urbanism attempts to do several things. Firstly, as discussed above, it seeks to centre an acknowledgement of the multiple temporal calibrations between endurance and expiration that shape urban spaces marked by international interventionism – something that does not feature prominently in the literature on humanitarian urbanism nor work on humanitarian enclaves or refugee camps in the same ways. Relatedly, and secondly, it foregrounds the relational geographies that are rooted in the Global South as both the site of most contemporary peacekeeping missions and the place from whence most peacekeepers on these missions come (Cunliffe, 2013). These relational geographies are as proximate as exchanges in Spanish between Uruguayan officers and neighbouring children in Goma and as distant as those between a soldier and his family in Uruguay.

Lastly, while it offers scholars the ability to distinguish between humanitarianism and military peacekeeping as it shapes urban spaces, it also seeks to create continuities across disciplines. I borrow the term ‘peace-kept’ from Jennings (2015), who uses it to refer to the target population of peacekeepers’ protection, qualify the cities where interactions between the two groups occur and describe how different actors navigate the economic opportunities in places where there is peacekeeping. Jennings uses the qualifier ‘peace-kept’ always in scare quotes, casting doubt onto the existence of a peace to be kept and upon the effectiveness of peacekeepers to fulfil such a mandate. Adopting this orthography is important to the notion of ‘peace-kept’ urbanism that I propose, as it does not represent an actual situation but a type of open and unfinished promise upon which the enterprise of multi-national military intervention is predicated. Moreover, were peace to be achieved in eastern DRC, the presence of peacekeepers and their attendant ways of living and building in Goma would ostensibly cease to exist. In order for the urban modes of making described in this article to occur, armed conflict and operational responses need to exist, but need to (and do) largely take place elsewhere, beyond the city.

## Conclusion

‘Peace-kept’ urbanism is a mode of making urban space with and in the presence of peacekeepers and a consideration of how various spatial and temporal horizons shape the built environment in places where there is peacekeeping. Everyday life in the ‘peace-kept’ places of the city and the camp are constantly moving back and forth between co-existing efforts to endure and expire that cannot be captured if we are not open to looking at trajectories beyond *in situ* settlement development over linear time.

The ‘more-than-linear’ relationships between time and the built environment brought to bear here problematise framings that view durability and settled modes of dwelling as necessary ingredients of urbanism and urbanity and as forces that can lessen precarity and uncertainty of temporary conditions. As the first example of the re-located Senegalese camp illustrates, temporal endurance does not necessarily lead to consolidation of urban forms in more permanent arrangements. Decisions about dwelling arrangements in urban areas must constantly grapple with the work of time on the land market and rising rents that shape the city. The two examples from the decades-old URUBATT camp highlight the mediating quality of camp space-time as one between active field deployments and a home base, as well as between the place of the mission and the places peacekeepers’ come from. Understanding the late emergence of such public spaces in the camp as the *Rincón del Hacha* brings into focus how new architectures and practices continue to emerge in enduring camps despite knowledge of an imminent expiration. Lastly, the planned endurance and consolidation of peacekeeping camps as more permanent features of the city and their re-use by national security forces may not preclude precarity, but stand to engender new forms of insecurity.

As the events illustrated in this article have shown, the contingencies of peacekeeping as it translates into a locative, dwelling practice offers an epistemological position from which we can uncover the multiple and more-than-linear temporalities through which urbanism and urban life can and do emerge. Temporal calibrations and defiances co-exist in the built environment of the peacekeeping camp, registering multiple elsewheres through which the camp is made – including the city just beyond its gates, field deployments in other parts of the DRC, as well as the homes from whence peacekeepers come and to which they plan to return. These elsewheres are tethered to synchronous elsewhens that impel physical changes and continuities in the camps that accommodate mobile, soldierly bodies. The movement of peacekeepers themselves, their arrivals and departures to and from the camp to proximate and distant places (with their attendant tempos and durations) are critical to the open-ended making and re-making of the camp with the spatio-temporal horizons that itinerant peacekeepers, commanders, mission leaders, and the city itself bring into view.

‘Peace-kept’ urbanism is a socio-spatial form begotten by experiences of encampment – although markedly different encampments than those that appear in camp studies literature. As Hailey (2008), writing in this literature, reminds us: ‘camping we experience time as duration and we reread place through our own itinerancy’ (p. 2). To better understand the transformations possible across temporal horizons of endurance and ephemerality, ‘peace-kept’ urbanism demands that we pair our analytical attention to dwelling with a dialectic of journeying to understand the temporal logics shaping the geographies and architectures of the places we inhabit, no matter the duration. ‘Peace-kept’ urbanism equally orients our attention, though never completely, to the interior spaces of dwelling among peacekeepers – a novel and open field for urban anthropological inquiry.

Too often, urban scholarship continues to overlook or devalue the place of the provisional in the production of urban spaces and lifeworlds despite attempts by scholars of southern urbanism and camp studies to recover its significance and possibilities. The temporariness of a transient urban settlement does not make it less urban. Rather its elasticity and provisionality can provide desirable social, economic and spatial alternatives that demand consideration in urban planning, project development and scholarly inquiry. The temporal condition of the peacekeeping camp and its inhabitation as an urban practice between dwelling and journeying reminds us to be wary of associating the temporal with the deficient and increasing permanence as promising a more certain future. Moreover, connections and considerations of elsewhere imagined at multiple scales are central to thinking about dwelling arrangements in their multiple temporal calibrations. What this means for Goma has less to do with the political possibilities of the peacekeepers themselves, but a ‘peace-kept’ population experiencing the city amidst protracted dislocation, and recognising what horizons fuel their connections and valuations of temporary settlement configurations.

## Notes

1. Congolese civilians not working for the mission notably enter different camps to attend meetings or skills courses convened by different military contingents.

2. My own access to the various peacekeeping camps in and around Goma was negotiated directly with the national contingents accommodated in such camps.

3. While this article presents a novel argument, some of the empirical material on which it relies appears in Chapter 5 of my book due for publication in 2025 (Larsen, forthcoming).

4. The necessity to make this distinction was inspired by the framing of the panel ‘Temporality and permanence of urbanization in Africa’ at the ECAS 2023: African Futures conference in Cologne, Germany. This panel was convened by Karen Büscher, Cristina Udelsmann Rodrigues and Patience Mususa.

5. I borrow the term ‘peace-kept’ from Kathleen Jennings (2015) and discuss its variable meanings later in this article.

6. Notable exceptions of work in which peacekeeping camp space features prominently in accounts of UN mission sites include Polman (2004), Higate and Henry (2009) and Henry (2015).

7. As Michael Pugh argues, peacekeeping is not humanitarianism. It is something that can be combined with or used in support of more purely humanitarian activities however (Pugh, 1996).

8. *Jambaar* means ‘courage’ in Wolof – the lingua franca of Senegal.

9. The Level III hospital is now a part of MONUSCO’s logistics base, located on 10 hectares of land owned by the Congolese *Regie des Voies Aériennes* (RVA).

10. *Mboq* means ‘corn’ in Wolof.

11. The Uruguayan battalion camp is adjacent to the new Senegalese FPU camp; the land is similarly owned by the state and is undevelopable based on its proximity to the runway.
